# Can the Use of Closed Incision Negative Pressure Wound Therapy in Immediate Prepectoral Breast Reconstruction With Polyurethane-Coated Implants Reduce the Rate of Early Complications?: A Comparative Study

**DOI:** 10.1093/asj/sjaf211

**Published:** 2025-10-23

**Authors:** Marzia Salgarello, Niccolò Lazzeri Domar, Giuseppe Visconti, Lorenzo Scardina, Sabatino D’Archi, Alba Di Leone, Gianluca Franceschini, Liliana Barone Adesi

## Abstract

**Background:**

Immediate prepectoral breast reconstruction with polyurethane-coated implants provides esthetic and functional benefits compared with subpectoral techniques, but early postoperative complications remain a concern. Closed incision negative pressure wound therapy (ciNPWT) has demonstrated benefits in other surgical fields, though evidence in breast reconstruction is limited.

**Objectives:**

To evaluate whether ciNPWT reduces early postoperative complications after immediate prepectoral reconstruction with polyurethane-coated implants, particularly in high-risk patients.

**Methods:**

We retrospectively analyzed 620 patients undergoing unilateral or bilateral conservative mastectomy with immediate prepectoral reconstruction using polyurethane-coated implants between May 2021 and February 2024. Patients were divided into 2 cohorts: 257 treated with ciNPWT and 363 with conventional dressings. Subgroup analyses considered mastectomy flap thickness (0.5-0.6 cm, 0.7-0.9 cm, ≥1.0 cm) and specimen weight (<500 g vs ≥500 g). Early complications (≤30 days) were classified as major (requiring reoperation or hospitalization) or minor.

**Results:**

ciNPWT significantly reduced major complications compared with controls: wound dehiscence (1.17% vs 6.61%), flap ischemia (0% vs 2.2%), major infection (0.39% vs 2.48%), and implant extrusion (0.39% vs 2.2%). Minor complications, including periprosthetic seromas, were also reduced (4.28% vs 5.79%). The protective effect was most evident in patients with thin mastectomy flaps (0.5-0.9 cm) and specimen weight ≥500 g. Correlation between intraoperative flap thickness and preoperative Rancati score confirmed benefits in high-risk patients (Rancati type 1).

**Conclusions:**

ciNPWT reduces early complications in immediate prepectoral reconstruction, with greatest benefit in high-risk patients. Integration into reconstructive protocols may enhance safety and broaden indications. Prospective randomized trials are warranted.

**Level of Evidence: 3 (Therapeutic):**

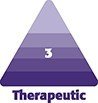

Breast cancer is the most common malignancy among women and the third overall, affecting approximately 1 in 8 women during their lifetime.^1^ Mastectomy can have severe psychological impacts,^2,3^ making reconstructive surgery a crucial component of treatment.^4^ However, adjuvant therapies such as radiotherapy often limit reconstructive options due to the increased risk of early complications like infection, flap failure, implant exposure, reconstruction loss and capsular contracture.^5-7^

Prepectoral implant-based reconstruction offers several advantages such as reduced morbidity and improved esthetic outcomes, compared to subpectoral approaches. Nonetheless, its broader adoption is limited by the risk of complications—especially in the early postoperative period—such as mastectomy flap ischemia, wound dehiscence, seroma and implant loss.^8^

Closed incision negative pressure wound therapy (ciNPWT) has demonstrated benefits in various surgical fields,^9^ promoting healing by reducing edema, improving perfusion, and limiting fluid accumulation.^10^ However, evidence supporting its use in prepectoral breast reconstruction, still remains underexplored.

This retrospective study examines 620 patients who underwent immediate prepectoral breast reconstruction with polyurethane-coated silicone implants after conservative mastectomy. Two cohorts were evaluated: 257 patients treated with ciNPWT using the PICO system (Smith & Nephew, London, UK), and 363 patients who received standard dressings (Steri-Strips and sterile gauzes). The objective was to assess whether ciNPWT reduces postoperative complications and improves healing outcomes, thereby increasing overall safety of prepectoral implant-based breast reconstruction.

To our knowledge, this is the largest study to date on ciNPWT in postmastectomy breast reconstruction and the only 1 evaluating the combined use of the PICO system (Smith & Nephew, London, UK) and prepectoral reconstruction with polyurethane-coated implants.

## METHODS

### Study Design

Between May 1, 2021, and February 9, 2024, a database was compiled, including all female patients who underwent unilateral or bilateral conservative mastectomy—specifically nipple-sparing (NSM), skin-sparing (SSM), and skin-reducing (SRM) mastectomy—followed by immediate prepectoral reconstruction with polyurethane-coated implants. No primary subpectoral direct-to-implant reconstructions were performed during the study period. Patients who underwent mastectomy without reconstruction were likewise excluded from this database, as they fell outside the scope of the current study. The database represented a consecutive series of patients.

A total of 620 patients were included, of whom 190 underwent bilateral procedures, resulting in 810 mastectomies available for analysis. The mean patient age was 51 years (range 18-88), and the mean body mass index (BMI) was 23.29 kg/m² (range 16.38-58.48). Tumor subtypes were distributed as follows: luminal A in 29.84% (185 patients), luminal B in 18.87% (117), HER2-positive luminal in 24.19% (150), HER2-positive non-luminal in 11.13% (69), and triple-negative in 15.97% (99).

No exclusion criteria were applied based on patient- or therapy-related risk factors. Consequently, patients with conditions such as age ≥65 years, Type I/II diabetes, BMI ≥ 30 kg/m², mastectomy specimen weight ≥500 g, vascular disorders, hypertension, immune-related diseases, hypersensitivity syndrome, smoking habits, polypharmacy, and preoperative (neoadjuvant) chemotherapy were included (Supplemental Table 1, available online at https://doi.org/10.1093/asj/sjaf211).

In accordance with current literature,^11-13^ certain risk factors were classified as major due to their well-documented impact on postoperative complications—specifically, flap thickness ≤0.5 cm, active smoking, and mastectomy specimen weight ≥500 g. Other factors, although potentially relevant, were considered minor given their less direct or less consistently demonstrated association with adverse outcomes. The only exclusion criteria were a documented allergy to any components of the PICO system (Smith & Nephew, London, UK) and a history of preoperative radiotherapy.

The study examines 2 cohorts of patients based on the time of treatment. The first cohort included 363 patients who underwent surgery between May 1, 2021, and December 15, 2022, and received standard wound dressings (Steri-Strips and sterile gauzes); this group is referred to as the “NO-ciNPWT cohort”. The second cohort included 257 patients treated between December 16, 2022, and February 9, 2024, who received ciNPWT using the PICO system (Smith & Nephew, London, UK); this group is referred to as the “ciNPWT cohort”. All other aspects of surgical management, reconstructive technique, and follow-up protocols were consistent between the 2 cohorts.

Both cohorts were further stratified into 3 subgroups based on mastectomy flap thickness measured at the incision site. The grouping was based on risk stratification for the development of short-term postoperative complications:

- Group 1: 0.5-0.6 cm (52 patients, 54 mastectomies)
- Group 2: 0.7-0.9 cm (253 patients, 303 mastectomies)
- Group 3: ≥1.0 cm (315 patients, 453 mastectomies)

Group 3 represents a low risk of postoperative complications, Group 2 a moderate risk, and Group 1 a high risk. The distribution of flap thickness groups according to ciNPWT use is detailed in Table 1.

**Table 1.** Distribution of the Study Population based on Group Assignment According to Mastectomy Flap Thickness Measured At the Incision Site

	NO-ciNPWT		ciNPWT	
Group 1 (0.5-0.6 cm)	25 patients	26 mastectomies	27 patients	28 mastectomies
Group 2 (0.7-0.9 cm)	160 patients	185 mastectomies	93 patients	118 mastectomies
Group 3 (≥1.0 cm)	178 patients	267 mastectomies	137 patients	186 mastectomies

In cases of bilateral mastectomy, group classification is determined by the side with the thinner mastectomy flap at the level of the incision site. ciNPWT, closed incision negative pressure wound therapy.

Patients were also stratified by mastectomy specimen weight, using 500 grams as a clinically relevant threshold for increased postoperative risk.^11,12^ In bilateral cases, patients were included in the “<500 g” group only if both mastectomy specimens were below this threshold; otherwise, they were classified in the “≥500 g” group. Of the 810 mastectomies, 58 exceeded this threshold, whereas 752 were <500 g. Direct-to-implant reconstruction was performed even in cases exceeding 500 g when intraoperative assessment confirmed adequate perfusion and patients expressed a preference for single-stage reconstruction. Nipple-sparing mastectomy was selected whenever oncological safety was confirmed by preoperative imaging and intraoperative frozen section analysis.

### Clinical Protocol and Surgical Technique

This study was conducted in accordance with the Declaration of Helsinki and with Articles 13 and 14 of the EU General Data Protection Regulation 2016/679. Institutional Review Board approval was obtained from the Ethics Committee “Lazio Area 3”. Written consent was provided, by which all patients agreed to the use and analysis of their data. All patients in this study were evaluated by a Multidisciplinary Surgery Board, which determined the most appropriate surgical strategy (breast conserving approach vs conservative mastectomy, NSM, SSM, or SRM) and reconstructive approach.^14^

Preoperative planning for conservative mastectomy included bilateral breast measurements (jugulum-nipple distance, nipple-inframammary fold distance, and nipple-nipple distance), assessment of ptosis (Regnault classification score^15^), and documentation of any asymmetries. Estimated implant volumes and mastectomy flap thickness (assessed by pinch test and mammography) were also recorded. Preoperative radiological assessment included evaluation of mastectomy flap thickness using the Rancati score,^16^ based on mammograms performed no earlier than 3 months before surgery.

Antibiotic prophylaxis consisted of a single intraoperative dose of 2 g Cefazolin. All mastectomies were performed by 4 senior breast surgeons using nipple-sparing (NSM, 724 mastectomies), skin-sparing (SSM, 70 mastectomies), or skin-reducing mastectomy (SRM, 16 mastectomies) techniques. In NSM cases, 92% of incisions (666 mastectomies) followed a radial pattern, whereas the remaining employed an inframammary approach. SRMs were performed using the inverted T technique, selected according to the degree of breast ptosis (based on Regnault's classification^15^), the presence of macromastia, and, when applicable, patient preference for breast volume reduction.

All patients underwent immediate prepectoral reconstruction using either hyper- or moderately projected anatomical silicone implants coated with polyurethane foam. Implant sizes were matched to or slightly exceeded mastectomy weights, as larger implants were considered to increase the risk of complications.

Suitability for prepectoral breast reconstruction was evaluated using the Rancati score,^16^ which assesses surgical feasibility based on preoperative mammographic imaging (Table 2) and by direct intraoperative measurement of mastectomy flap thickness (see Table 1, showing the distribution of mastectomy flaps thickness at the incision site in this series). According to the Rancati score, patients with Type 1 flaps (thickness < 1 cm) are generally considered unsuitable for prepectoral reconstruction.^16^ In our protocol, however, no patient was excluded solely on this basis. Intraoperatively, flap perfusion was systematically assessed using indocyanine green (ICG) angiography. If vascularization was adequate, prepectoral implant placement was performed in flaps with a thickness < 1 cm; conversely, in cases of insufficient perfusion, reconstruction was converted to a 2-stage sub-muscular approach with temporary tissue expander placement; such cases were not included in the present analysis, since they received standard dressings without ciNPWT. Over the study period, this occurred in 12 patients, who would have been classified within thickness Group 1.

**Table 2.** Rancati Score in Our Series of 810 Mastectomies

Classification	Size (cm)	No. of mastectomies
Type 1 (poor coverage)	<1	130
Type 2 (medium coverage)	1-2	537
*Type 3 (good coverage)*	>2	143

Following surgical steps included adjustment of the mastectomy pocket and meticulous hemostasis. One or 2 15 Fr J-VAC drains were placed within the pocket and maintained under suction. Implants were irrigated with 10 cc of rifamycin (250 mg/3 mL), and prior to implantation, the surgical field was disinfected with 10% povidone-iodine (Betadine, povidone-iodine, Mundipharma, Cambridge, UK) and surgeons changed gloves. Implants were inserted with attention to optimal positioning, verified in a semi-seated position. Closure was performed in layers using absorbable 3/0, 4/0, and 4/0 Rapide Vicryl sutures (Ethicon, Somerville, NJ, USA).

In the ciNPWT cohort, the skin was rinsed with saline, degreased using an alcohol-based solution (CITROSIL, benzalkonium chloride, Angelini Pharma, Rome, Italy), and a sterile PICO system (Smith & Nephew, London, UK) was applied. In contrast, the NO-ciNPWT cohort received conventional postoperative dressing with Steri-Strips and sterile gauzes.

### Postoperative Protocol

The average hospital stay was 2-3 days. After discharge, patients underwent routine follow-up visits twice weekly for 3 weeks to monitor for postoperative complications, including wound dehiscence, mastectomy flap necrosis, seroma, site infection, systemic infection, and fever.

The ciNPWT system was maintained in place for 7 days, in line with the manufacturer's protocol. This timeframe covers the critical first postoperative week, corresponding to early wound healing. Although no direct comparison with shorter or longer application exists, the physiologic rationale is to provide continuous support until flap adherence and early re-epithelialization are achieved. Drains were removed when daily output decreased to less than 20 cc per day, to reduce the risk of seroma formation, which could compromise implant adhesion to surrounding tissues and impair the benefits of polyurethane-coated implants; seromas observed in our series are reported in Table 3.^17,18^ A few days after drain removal, a routine follow-up ultrasound was performed to detect any periprosthetic fluid collections. At the 14-day postoperative checkup, sutures were removed, and patients were allowed to resume full showering. Return to regular daily activities and, subsequently, physical activity occurred approximately 1 month after surgery.

**Table 3.** Overall Complication Rates Stratified By ciNPWT System Use and Mastectomy Flap Thickness At the Incision Site

Complication	Total NO ciNPWT (363 pts)	Total ciNPWT (257 pts)	Group 1 + NO ciNPWT (25 pts)	Group 1 + ciNPWT (27 pts)	Group 2 + NO ciNPWT (160 pts)	Group 2 + ciNPWT (93 pts)	Group 3 + NO ciNPWT (178 pts)	Group 3 + ciNPWT (137 pts)
Wound dehiscence requiring revision	24 patients (6.61%)	3 patients (1.17%)	12 patients (48%)	3 patients (11.1%)	5 patients (3.13%)	0 patients	7 patients (3.93%)	0 patients
Ischemic suffering of mastectomy flaps requiring revision	8 patients (2.2%)	0 patients	4 patients (16%)	0 patients	3 patients (1.88%)	0 patients	1 patient (0.56%)	0 patients
Major site infection	9 patients (2.48%)	1 patient (0.39%)	1 patient (4%)	0 patients	4 patients (2.5%)	1 patient (1.08%)	4 patients (2.25%)	0 patients
Implant extrusion	8 patients (2.2%)	1 patient (0.39%)	4 patients (16%)	0 patients	3 patients (1.88%)	1 patient (1.08%)	1 patient (0.56%)	0 patients
Minor complications	21 patients (5.79%)	11 patients (4.28%)	5 patients (20%)	3 patient (11.1%)	7 patients (4.38%)	4 patients (4.3%)	9 patients (5.06%)	4 patients (2.92%)
Periprosthetic seromas	6 patients (1.65%)	1 patient (0.39%)	2 patients (8%)	1 patient (3.7%)	3 patients (1.88%)	0 patients	1 patient (0.56%)	0 patients

“Major site infection” refers to infections that required hospital admission for systemic antibiotic therapy and were therefore classified as major complications. ciNPWT, closed incision negative pressure wound therapy.

### Outcome Measures

The primary outcome was the incidence of short-term complications (within 30 days postoperatively), classified as either major or minor. Major complications were defined as those requiring surgical intervention, systemic pharmacologic therapy, or hospital admission. Minor complications included events that did not require systemic pharmacological therapy, surgical intervention, or hospital admission (such as seromas managed conservatively or via aspiration, minor ischemia not requiring revision, and superficial wound dehiscence treated with extended dressing care alone). Specifically, the complications assessed included hematoma, seroma, ischemic compromise of the mastectomy flaps, wound dehiscence, site infection, and implant exposure. The objective was to evaluate the postoperative complication rate associated with prepectoral implant-based breast reconstruction, comparing the 2 patient cohorts to determine whether ciNPWT improves safety and reduces reoperation rates.

## RESULTS

Patients had a mean age of 51 years (range 18-88). The average mastectomy specimen weight was 240 g (range: 40-1340 g), the average implant volume was 265 cc (range: 95-680 cc), and the average mastectomy flap thickness at the incision site was 1 cm (range: 0.5-2.5 cm). The mean follow-up period was 24 months, ranging from a minimum of 12 to a maximum of 48 months. A breast weight ≥500 g,^11,12^ a mastectomy flap thickness ≤0.6 cm, and a positive smoking history^19^ were all associated with an increased risk of postoperative complications.

Short-term complications (within 30 days postoperatively) were systematically recorded and analyzed based on whether patients received ciNPWT or conventional dressings, as detailed in Table 3. A marked difference in complication rates was observed between patients in Groups 1 and 2 and those with thicker flaps (≥1.0 cm, Group 3) in both the ciNPWT and NO-ciNPWT cohorts (Table 3). Notably, the use of ciNPWT consistently reduced complication rates across all 3 flap thickness groups. The most substantial reductions were observed in patients with thinner flaps (0.5-0.9 cm measured intraoperatively), in whom ciNPWT yielded the most pronounced benefit.

Correlation analysis between intraoperative flap thickness and preoperative mammographic assessment (Rancati score,^16^ Table 4) showed that 38.89% of cases in Group 1 and 22.67% of cases in Group 2 corresponded to Rancati Type 1. Patients with thicker flaps (≥1.0 cm), who in our series were predominantly classified as Rancati Type 2 (68.43%) and Type 3 (22.74%), also experienced favorable outcomes with ciNPWT, albeit with less marked differences compared to controls. Overall, in our series of 810 mastectomies, preoperative radiological assessment according to the Rancati score^16^ tended to slightly overestimate, as shown by our data, the clinical flap thickness later measured intraoperatively at the incision site.

**Table 4.** Correlation Between Mastectomy Group Assignment based on Intraoperative Mastectomy Flap Thickness Measured At the Incision Site and Preoperative Rancati Classification, Assessed on Mammograms Performed Within 3 Months Prior to Surgery

Intraoperative flap thickness (groups)	Rancati Type 1 (<1 cm)	Rancati Type 2 (1-2 cm)	Rancati Type 3 (>2 cm)	Total mastectomies
Group 1 (0.5-0.6 cm)	21 (38.89%)	33 (61.11%)	0	54
Group 2 (0.7-0.9 cm)	69 (22.77%)	194 (64.03%)	40 (13.2%)	303
Group 3 (≥1 cm)	40 (8.83%)	310 (68.43%)	103 (22.74%)	453

**Figure 1.**
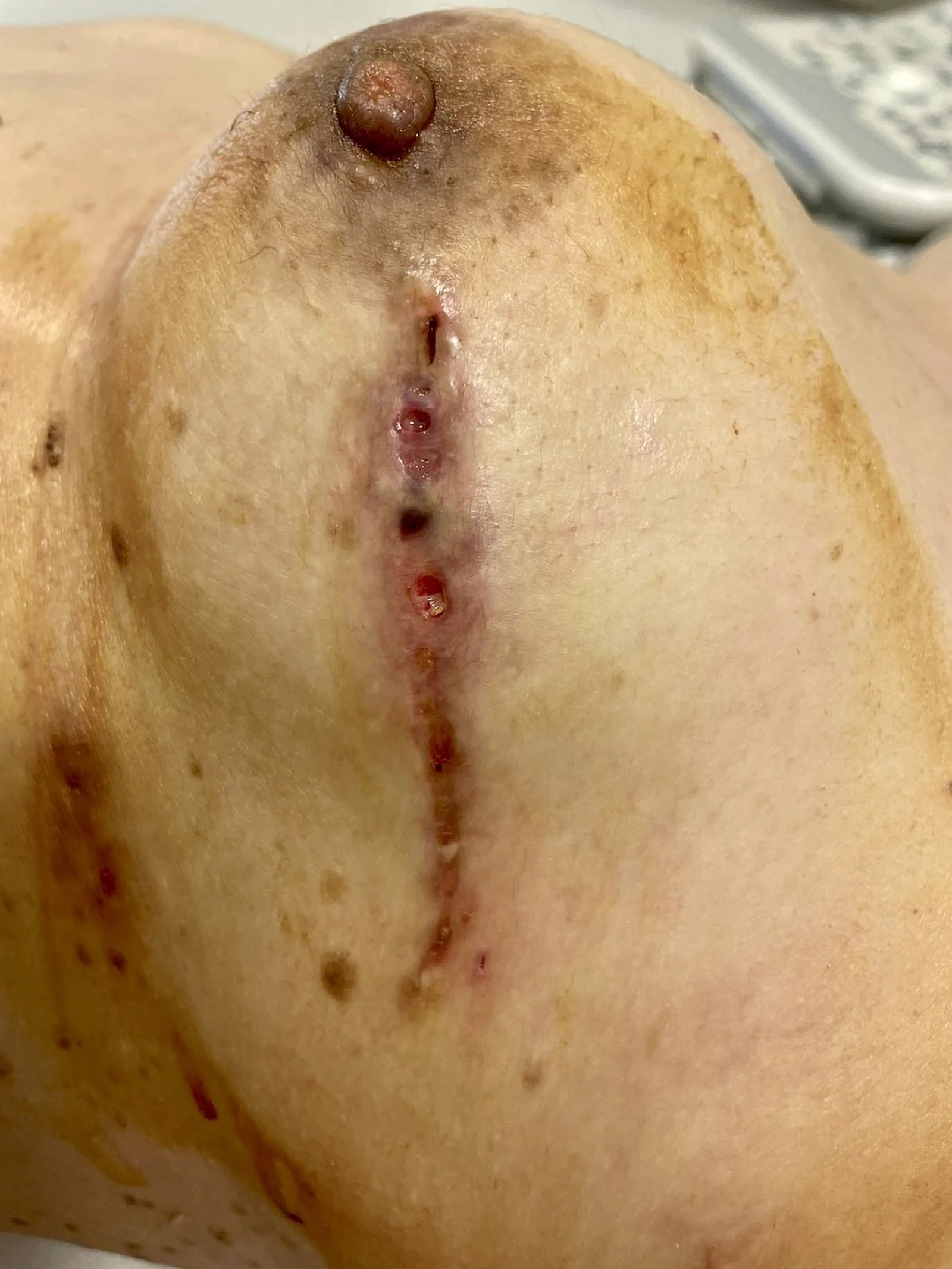
Wound dehiscence in a 49-year-old female patient who did not receive closed incision negative pressure wound therapy (ciNPWT). The breakdown along the mastectomy incision exposes the underlying implant.

The most substantial decreases associated with ciNPWT use were seen in major complications—those requiring surgical intervention or hospitalization (Table 3). Specifically, wound dehiscence requiring surgical revision decreased from 6.61% to 1.17% (Figure 1), flap ischemia from 2.2% to 0%, site infection from 2.48% to 0.39%, and implant extrusion from 2.2% to 0.39%. “Minor complications” also showed a reduction, though to a lesser extent (from 5.79% to 4.28%). Statistical analysis using Chi-square test and Fisher's exact test confirmed that the reduction in major complications in the ciNPWT cohort was significant (13.49% vs 1.95%, *P* < .001). Conversely, the difference in minor complications (5.79% vs 4.28%) did not reach statistical significance (*P* = .52).

**Figure 2.**
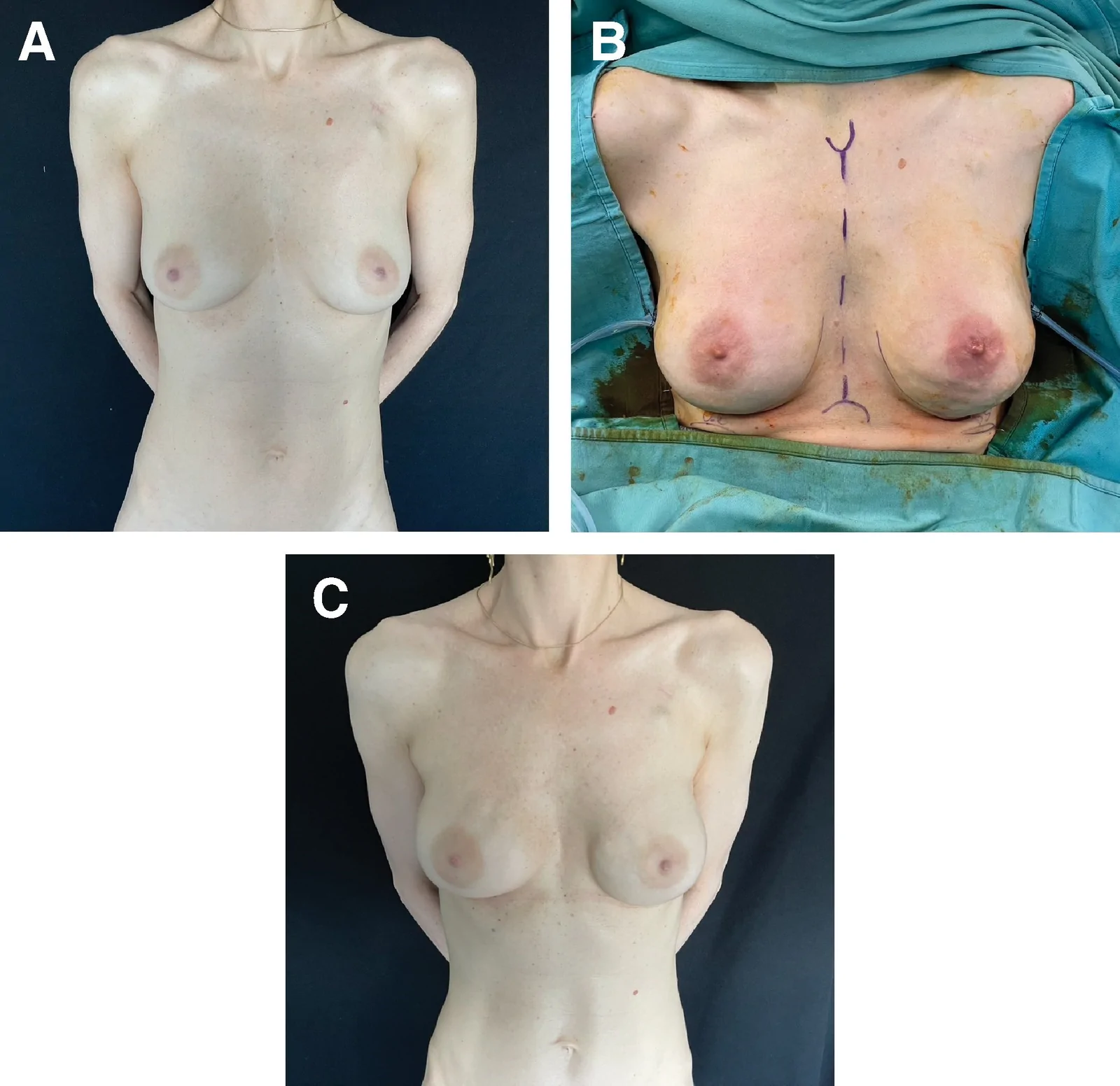
Bilateral mastectomy via inframammary approach in a 43-year-old female patient with breast cancer of Group 1 (flap thickness 0.5-0.6 cm) treated with standard dressings, no ciNPWT. (A) Preoperative frontal view. (B) Intraoperative view following mastectomy (specimen weight is 172 g on the right; 180 g on the left) and implant placement (Polytech, Dieburg, Germany, 30746, 265 mL bilaterally). (C) Postoperative frontal view at 8 months.

**Figure 3.**
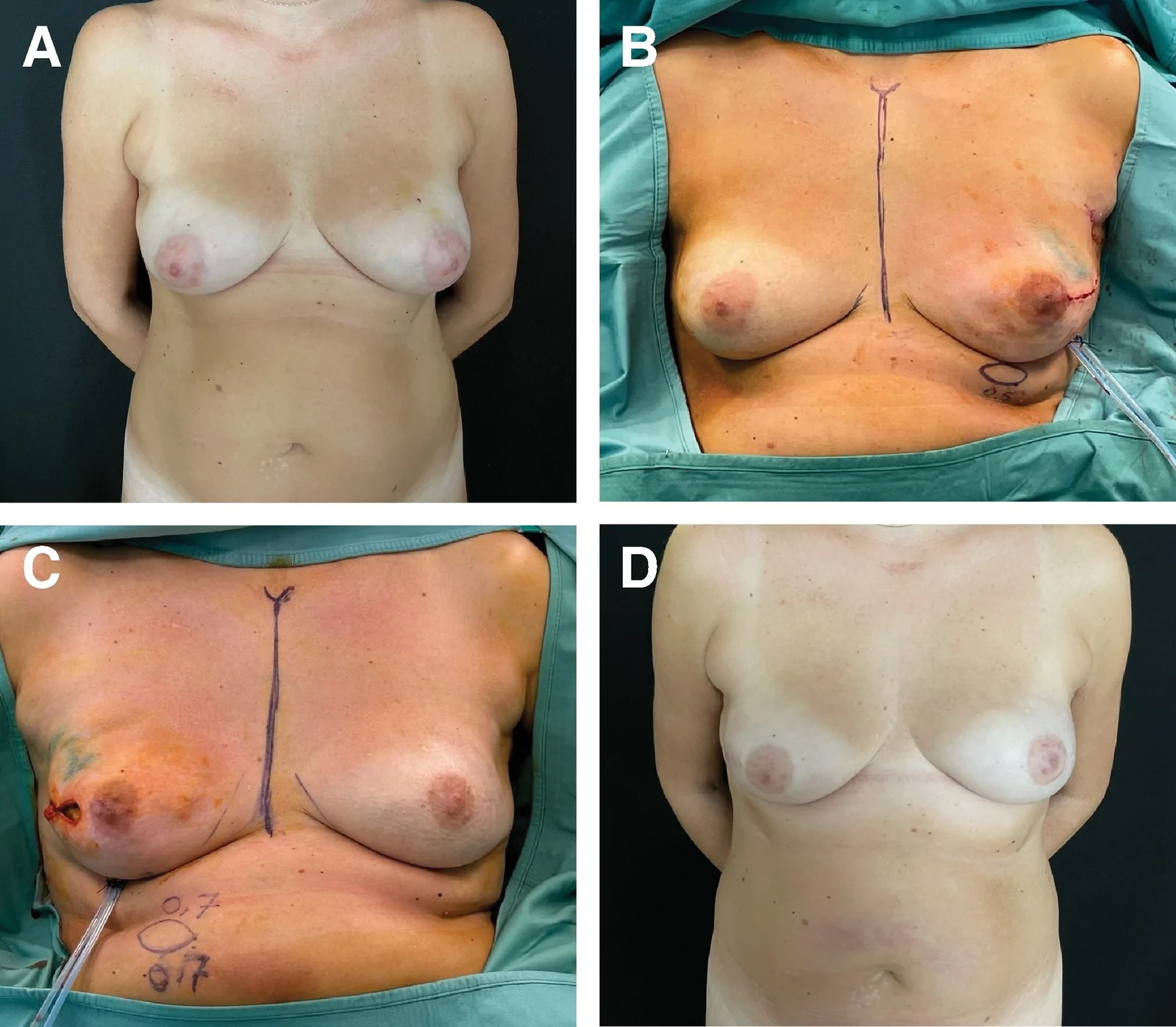
Bilateral mastectomy with lateral radial incisions in a 50-year-old female patient with breast cancer of Group 1 (flap thickness 0.5-0.6 cm) treated with closed incision Negative Pressure Wound Therapy (ciNPWT). Left mastectomy was performed first, and the contralateral procedure carried out 2 years later. (A) Preoperative frontal view. (B, C) Intraoperative view following mastectomy (320 g right specimen; 308 g left specimen) and implant placement (Polytech 30746, 295 mL on both sides). (D) Postoperative frontal view at 12 months from the right mastectomy.

**Figure 4.**
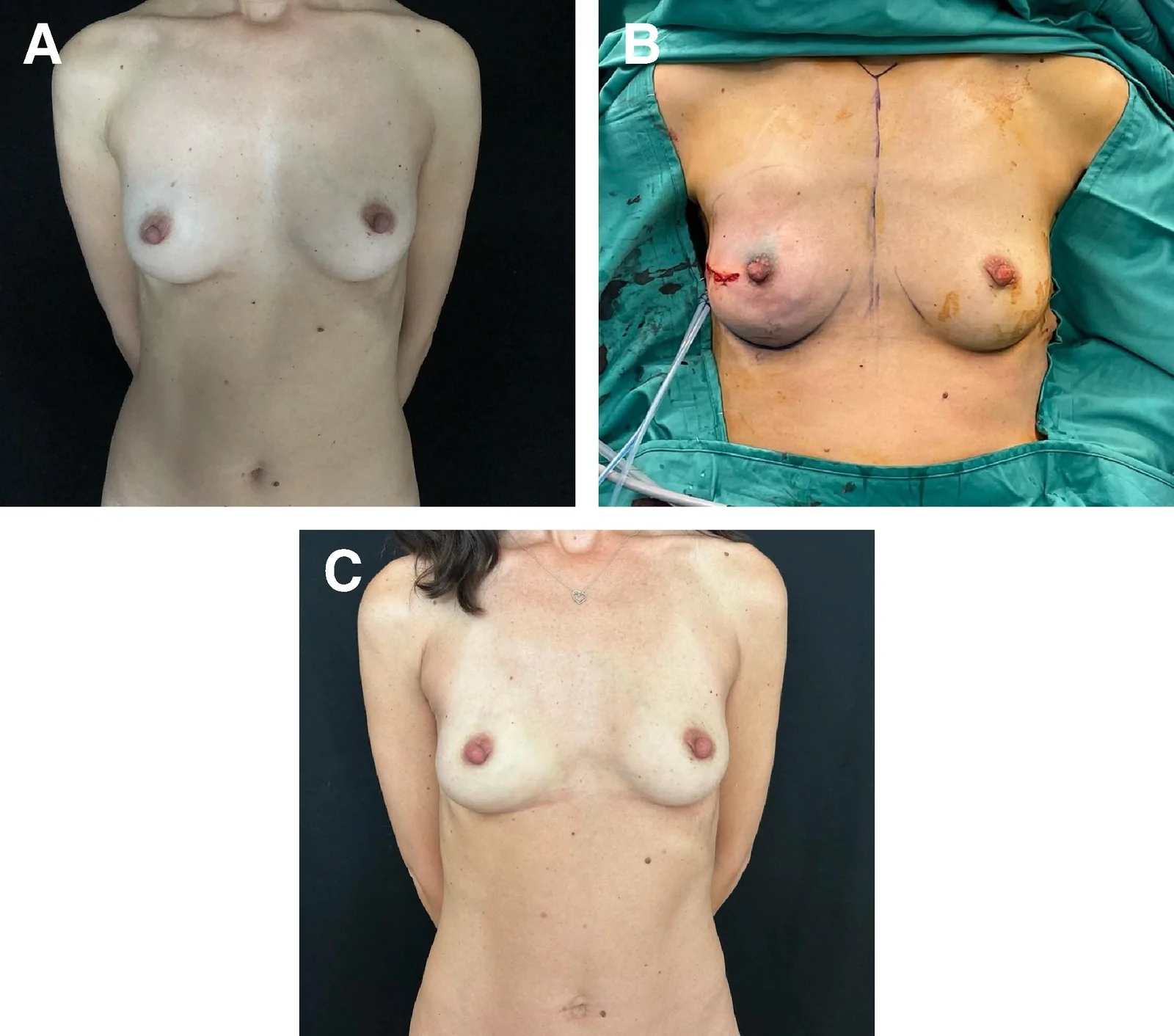
Right mastectomy with lateral radial incision in a 41-year-old female patient with breast cancer of Group 2 (flap thickness 0.7-0.9 cm) treated with standard dressings, no ciNPWT. (A) Preoperative frontal view. (B) Intraoperative view following mastectomy (145 g) and implant placement (Polytech 30745, 165 mL). (C) Postoperative frontal view at 2 years.

**Figure 5.**
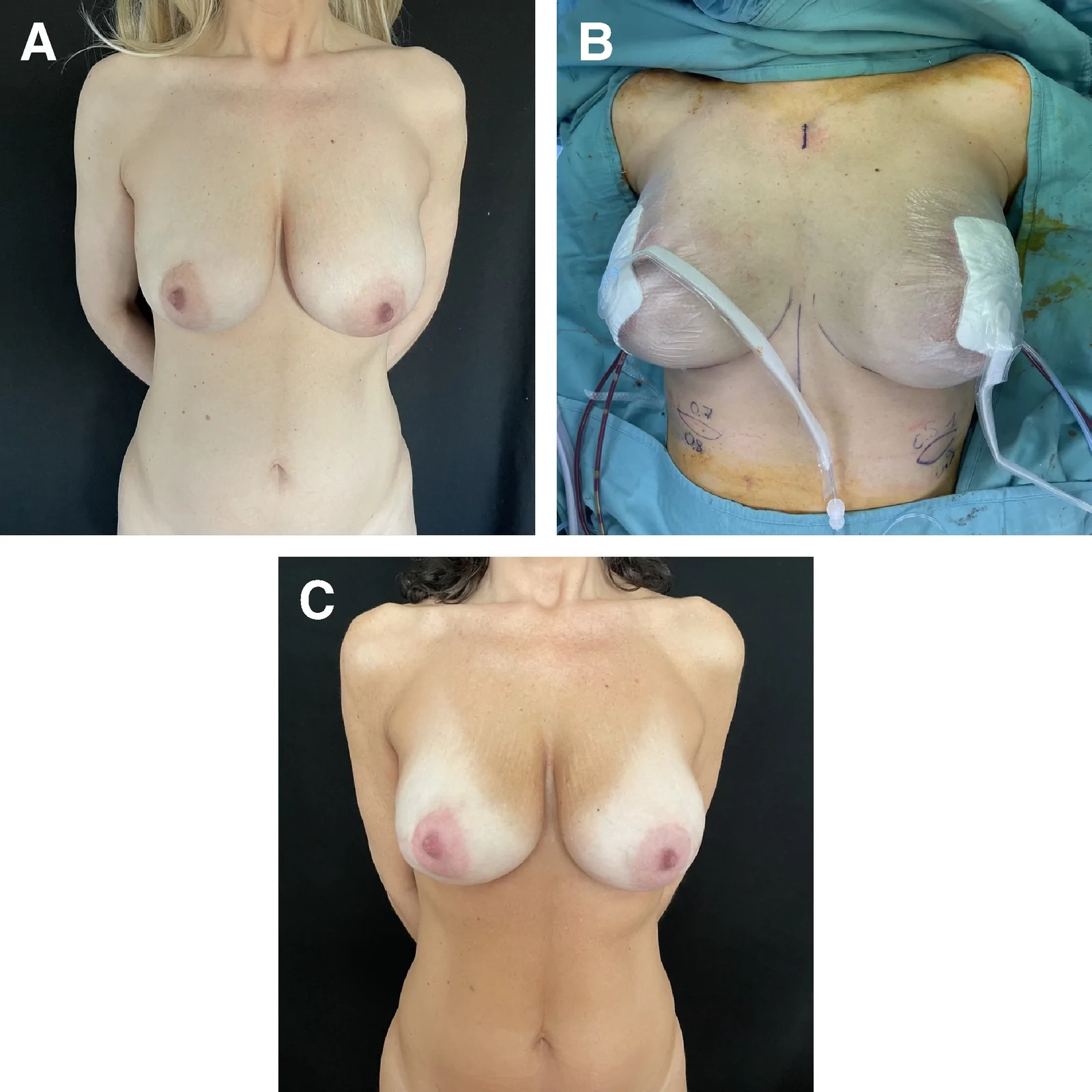
Bilateral mastectomy with lateral radial incisions in a 48-year-old female patient with breast cancer of Group 2 (flap thickness 0.7-0.9 cm) treated with closed incision Negative Pressure Wound Therapy (ciNPWT). (A) Preoperative frontal view. (B) Intraoperative view following mastectomy (150 g right specimen; 175 g left specimen) and implant placement (Polytech 30746, 295 cc on both sides), showing the placement of the ciNPWT system immediately after wounds closure. (C) Postoperative frontal view at 18 months.

**Figure 6.**
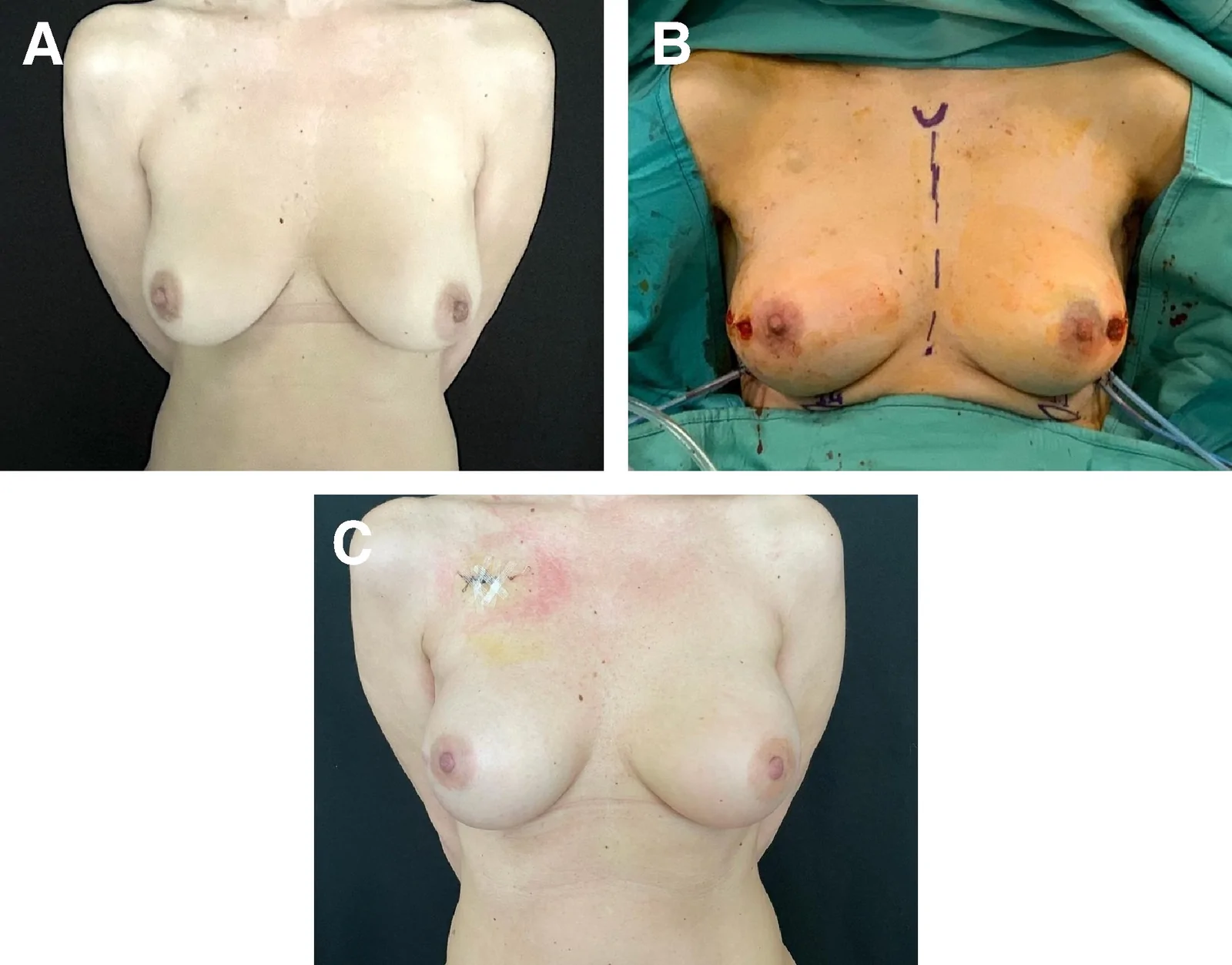
Bilateral mastectomy with lateral radial incisions in a 44-year-old female patient with breast cancer of Group 3 (flap thickness ≥1.0 cm) treated with standard dressings, no ciNPWT. (A) Preoperative frontal view. (B) Intraoperative view after mastectomy (153 g right specimen; 142 g left specimen) and implant placement (Polytech 30746, 230 cc bilaterally). (C) Postoperative frontal view at 16 months.

**Figure 7.**
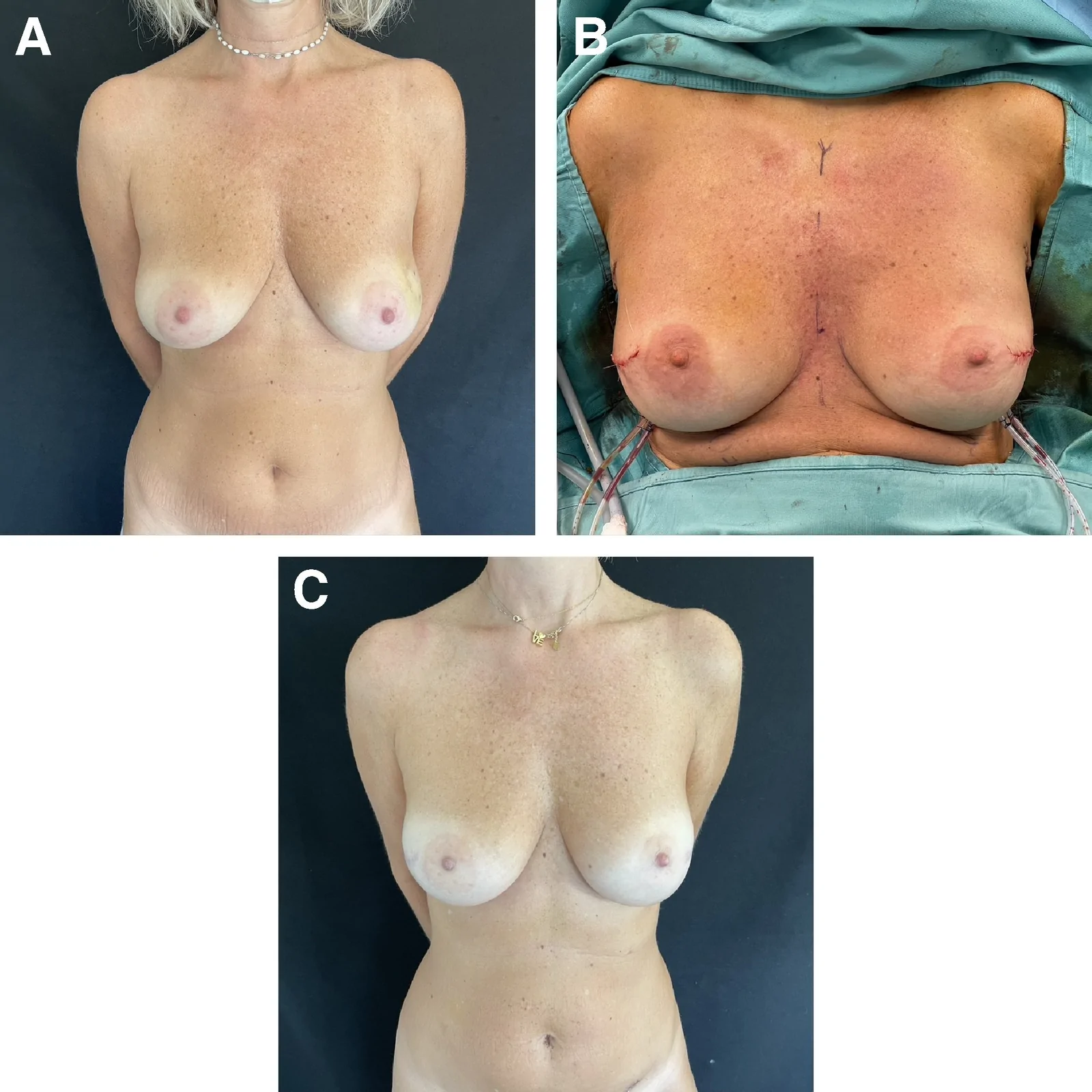
Bilateral mastectomy with lateral radial incisions in a 47-year-old female patient with breast cancer of Group 3 (flap thickness ≥1.0 cm) treated with closed incision Negative Pressure Wound Therapy (ciNPWT). (A) Preoperative frontal view. (B) Intraoperative view following mastectomy (250 g right specimen; 284 g left specimen) and implant placement (Polytech 30746, 335 cc bilaterally). (C) Postoperative frontal view at 12 months.

As shown in Table 5, mastectomy specimens weighing ≥500 g were associated with higher rates of both major and minor complications. Nevertheless, within both weight-based subgroups, the ciNPWT cohort consistently exhibited lower complication rates compared to the NO-ciNPWT group. When stratified by specimen weight, ciNPWT significantly reduced major complications in both <500 g (11.73% vs 1.72%, *P* < .001) and ≥500 g subgroups (40.92% vs 4.0%, *P* = .006). Differences in minor complications were not significant. Representative clinical cases are presented to illustrate the outcomes of patients across the different flap thickness groups, both without and with ciNPWT (Figures 2-7).

**Table 5.** Complication Rates Stratified By ciNPWT System Use and Mastectomy Weight

Complication	NO-ciNPWT < 500 g (341 pts)	ciNPWT < 500 g (232 pts)	NO-ciNPWT ≥ 500 g (22 pts)	ciNPWT ≥ 500 g (25 pts)
Wound dehiscence requiring revision	19 patients (5.57%)	2 patients (0.86%)	5 patients (22.72%)	1 patient (4%)
Ischemic suffering of mastectomy flaps requiring revision	6 patients (1.76%)	0 patients	2 patients (9.1%)	0 patients
Major site infection	8 patients (2.35%)	1 patient (0.43%)	1 patient (4.55%)	0 patients
Implant extrusion	7 patients (2.05%)	1 patient (0.43%)	1 patient (4.55%)	0 patients
Minor complications	17 patients (4.99%)	9 patients (3.88%)	4 patients (18.18%)	2 patients (8%)

“Major site infection” refers to infections that required hospital admission for systemic antibiotic therapy and were therefore classified as major complications. ciNPWT, closed incision negative pressure wound therapy.

## DISCUSSION

Breast cancer is the most frequent neoplasm among women and ranks third overall, with an estimated lifetime risk of 1 in 8 women.^1^ Surgical options include conservative procedures (lumpectomy or quadrantectomy, usually combined with radiotherapy) and mastectomy, both offering comparable survival rates.^13^ However, mastectomy is often preferred in specific cases, including patients under 40 years of age, BRCA mutation carriers, tumors ≥5 cm, multifocal lesions, or high risk of recurrence.^2,20,21^ Unilateral or bilateral mastectomy inevitably carries psychological consequences,^2,3^ underlining the importance of breast reconstruction in mitigating emotional distress.^4^

Reconstructive options include autologous and implant-based techniques, both of which may be performed immediately or in a delayed setting. The choice depends on various factors, including patient preferences, donor site availability, and the need to avoid delays in adjuvant oncologic therapies such as chemotherapy. Although anatomical suitability and patient satisfaction guide the choice between autologous and implant-based reconstruction, minimizing postoperative complications is essential, as these may delay systemic therapy and negatively impact oncologic outcomes.^5,22-24^ In the absence of planned postoperative radiotherapy, immediate implant-based reconstruction is generally preferred due to reduced cost, faster recovery, and the absence of donor-site morbidity.^25^

Implants can be positioned in either a retropectoral or prepectoral plane. Retropectoral placement is associated with drawbacks such as animation deformity, increased postoperative pain, and prolonged recovery.^8^ Although acellular dermal matrices have improved postoperative outcomes, their use has been linked to increased rates of seroma and infection; this is the main reason why acellular dermal matrices or synthetic meshes were not used in this series.^8,26,27^

Conversely, prepectoral breast reconstruction avoids animation deformity, reduces morbidity, and generally yields improved esthetic outcomes. However, it is not without disadvantages, and the literature on postoperative complications following immediate or delayed reconstruction remains conflicting.^5,28-30^ The use of polyurethane-coated implants has been proposed to enhance outcomes by minimizing malposition, rotation, seroma formation, and capsular contracture through improved tissue integration.^31,32^ The low incidence of periprosthetic seroma and wound dehiscence observed in our series may also be attributed to the mechanical and biological advantages of polyurethane-coated implants. The so-called velcro effect of polyurethane facilitates stable adhesion between the mastectomy flap and the implant shell. By enhancing flap stability, reducing shear forces at the wound edges, and minimizing dead space, polyurethane coating is thought to lower the risk of periprosthetic seroma formation^17,31^ and decrease tension along the incision line, thereby reducing the incidence of wound dehiscence.

Postoperative complications can compromise cosmetic results, lower patient satisfaction, and increase healthcare costs.^28,33^ The economic burden of such complications is significant and should not be underestimated.^34,35^ Infections represent one of the most serious complications, occurring at approximately twice the rate observed in mastectomy without reconstruction and at much higher rates than in augmentation mammoplasty.^30,36^ Known risk factors include obesity, diabetes, smoking, prolonged surgical time, and corticosteroid use.^9,19,30^

Site infections may lead to wound dehiscence and, in severe cases, reconstruction failure requiring implant explantation. Jeevan et al reported that approximately 9% of reconstructed patients ultimately require implant removal.^37^ Furthermore, increasing evidence links local and systemic inflammation to oncogenesis and cancer recurrence.^5^

negative pressure wound therapy (NPWT) was first introduced in the 1990s^38^ for the management of complex wounds via continuous sub-atmospheric pressure applied to the wound bed.^10^ Its mechanisms of action include improved perfusion (neoangiogenesis), granulation tissue formation, reduced wound exudate and mechanical tension, bacterial load control, and improved lymphatic drainage.^10,39^ NPWT also affects molecular pathways, upregulating VEGF, FGF-2, IL-6, IL-8, and modulating metalloproteinases,^40,41^ thereby accelerating healing and improving scar quality.^40^

In recent years, ciNPWT has been introduced in breast reconstruction surgery as a preventive therapy to reduce local complications,^9^ such as infection, seroma, necrosis, and wound dehiscence, with potential cost-saving implications.^10^ Various protocols regarding pressure levels, application duration, and outcome measures have been proposed,^42-44^ all reporting favorable results. However, no study to date has investigated the combined use of ciNPWT and polyurethane-coated implants in prepectoral breast reconstruction.

Our findings are in line with previous reports demonstrating the protective role of ciNPWT in implant-based breast reconstruction. Gabriel et al^42^ observed a reduction in wound complications and implant loss with ciNPWT compared to standard dressings, whereas Irwin et al^43^ specifically confirmed its benefit in prepectoral reconstructions. More recently, Cagney et al^10^ in a meta-analysis highlighted decreased surgical site complications with prophylactic ciNPWT across different breast surgery settings. Compared to these studies, our series is the largest to date and uniquely focused on prepectoral reconstruction with polyurethane-coated implants, suggesting a synergistic effect between the biological integration properties of polyurethane and the wound stabilization provided by ciNPWT.

The ciNPWT device (PICO system, Smith & Nephew, London, UK) was associated with a reduction in early postoperative complications across all patient subgroups, regardless of mastectomy flap thickness or specimen weight. The effect was especially notable in patients with thin mastectomy flaps (Groups 1 and 2) and in those with mastectomy weights ≥500 g, supporting broader indications for prepectoral reconstruction.

Most complications occurred within 30 days postoperatively and were more frequent in the NO-ciNPWT cohort. The most common adverse events were wound dehiscence requiring revision and site infection—both of which were significantly mitigated by ciNPWT through wound edge stabilization and sealed negative pressure therapy.

Importantly, although patients with Rancati score type 1 (<1 cm) are traditionally considered poor candidates for prepectoral reconstruction,^16^ our findings suggest that—with careful intraoperative assessment and the use of adjunctive measures such as ciNPWT and polyurethane-coated implants—even these high-risk patients can achieve safe and successful outcomes. This supports a more individualized, surgeon-led decision-making process rather than rigid exclusion criteria.

All cases underwent intraoperative assessment of flap vascularization with ICG angiography. Although robust perfusion analysis helps select suitable candidates, ciNPWT acts through complementary mechanisms such as improved perfusion (neoangiogenesis), granulation tissue formation, reduced wound exudate and mechanical tension, thereby providing additional benefits beyond perfusion assessment alone.

Patient tolerance of the device was generally favorable. No malfunctions of the suction unit were reported, removal was well tolerated, and complaints related to noise or discomfort were rare. However, patient-reported outcomes were not systematically assessed, representing a limitation of the present study. Additional limitations included its retrospective and non-randomized design, and the fact that it was a single-center study performed by a limited number of senior surgeons, potentially reducing the generalizability of the findings. Finally, cost-effectiveness analyses were not systematically conducted, limiting the ability to fully assess the health-economic value of ciNPWT in this setting.

## CONCLUSIONS

This study demonstrates that the use of ciNPWT with the PICO system (Smith & Nephew, London, UK) in immediate prepectoral breast reconstruction using polyurethane-coated silicone implants is associated with a significant reduction in short-term postoperative complications, including wound dehiscence, ischemic compromise of the mastectomy flaps, site infection, and implant extrusion. These findings were observed consistently across all patient subgroups, regardless of mastectomy flap thickness or mastectomy specimen weight, with the greatest impact seen in patients with thinner flaps (<1 cm) and mastectomy weights ≥500 g.

The ciNPWT system contributed to improved wound management and may have facilitated faster healing, although esthetic outcomes were not formally assessed. Importantly, ciNPWT appears to enhance the safety of prepectoral reconstruction even in patients traditionally considered high-risk—such as those with a history of smoking or elevated BMI—who are otherwise more prone to complications. Our findings suggest that the protective and healing-promoting effects of ciNPWT may mitigate these risks, thereby enabling safer implant-based reconstruction and potentially expanding eligibility for this surgical approach.

While the retrospective and non-randomized design represents a limitation, the results provide compelling evidence supporting the integration of ciNPWT into implant-based prepectoral breast reconstruction protocols. Future prospective, randomized controlled trials are warranted to confirm these findings and further refine patient selection criteria, treatment protocols, and cost-effectiveness analyses.

## Supplemental Material

This article contains supplemental material located online at https://doi.org/10.1093/asj/sjaf211.

## Supplementary Material

sjaf211_Supplementary_Data

## References

[1] Harbeck N, Gnant M. Breast cancer. Lancet. 2017;389:1134–1150. doi: 10.1016/S0140-6736(16)31891-8 27865536

[2] Fang SY, Shu BC, Chang YJ. The effect of breast reconstruction surgery on body image among women after mastectomy: a meta-analysis. Breast Cancer Res Treat. 2013;137:13–21. doi: 10.1007/s10549-012-2349-1 23225142

[3] Heimes AS, Stewen K, Hasenburg A. Psychosocial aspects of immediate versus delayed breast reconstruction. Breast Care. 2017;12:374–377. doi: 10.1159/000485234 29456468 PMC5803679

[4] Wilkins EG, Cederna PS, Lowery JC, et al Prospective analysis of psychosocial outcomes in breast reconstruction: one-year postoperative results from the Michigan breast reconstruction outcome study. Plast Reconstr Surg. 2000;106:1014–1025. doi: 10.1097/00006534-200010000-00010 11039373

[5] Beecher SM, O'Leary DP, McLaughlin R, Sweeney KJ, Kerin MJ. Influence of complications following immediate breast reconstruction on breast cancer recurrence rates. Br J Surg. 2016;103:391–398. doi: 10.1002/bjs.10068 26891211

[6] Karami RA, Ghanem OA, Ibrahim AE. Radiotherapy and breast reconstruction: a narrative review. Ann Breast Surg. 2020;4:17–17. doi: 10.21037/abs-20-71

[7] Zhu M, Mao J, Fang J, Chen D. Risk factors for severe complications and salvage management in direct-to-implant immediate breast reconstruction: a retrospective study. Medicine (Baltimore). 2024;103:e37365. doi: 10.1097/MD.0000000000037365 38457600 PMC10919468

[8] Wagner RD, Braun TL, Zhu H, Winocour S. A systematic review of complications in prepectoral breast reconstruction. J Plast Reconstr Aesthet Surg. 2019;72:1051–1059. doi: 10.1016/j.bjps.2019.04.005 31076195

[9] Willy C, Agarwal A, Andersen CA, et al Closed incision negative pressure therapy: international multidisciplinary consensus recommendations. Int Wound J. 2017;14:385–398. doi: 10.1111/iwj.12612 27170231 PMC7949983

[10] Cagney D, Simmons L, O’Leary DP, et al The efficacy of prophylactic negative pressure wound therapy for closed incisions in breast surgery: a systematic review and meta-analysis. World J Surg. 2020;44:1526–1537. doi: 10.1007/s00268-019-05335-x 31900568

[11] Chattha A, Bucknor A, Kamali P, et al Comparison of risk factors and complications in patients by stratified mastectomy weight: an institutional review of 1041 consecutive cases. J Surg Oncol. 2017;116:811–818. doi: 10.1002/jso.24753 28833196

[12] Kim MJ, Lee WB, Lee IJ, Hahn HM, Thai DQ, Kim JY. The investigation of the relation between expansion strategy and outcomes of two-stage expander-implant breast reconstruction. Gland Surg. 2022;11:1–11. doi: 10.21037/gs-21-515 35242664 PMC8825526

[13] Early Breast Cancer Trialists' Collaborative Group . Effects of radiotherapy and surgery in early breast cancer—an overview of the randomized trials. N Engl J Med. 1995;333:1444–1456. doi: 10.1056/NEJM199511303332202 7477144

[14] Barone Adesi L, Salgarello M, Di Leone A, et al Personalizing breast cancer surgery: harnessing the power of ROME (radiological and oncoplastic multidisciplinary evaluation). J Pers Med. 2025;15:114. doi: 10.3390/jpm15030114 40137430 PMC11943295

[15] Regnault P . Breast ptosis. Definition and treatment. Clin Plast Surg. 1976;3:193–203. doi: 10.1016/S0094-1298(20)30220-0 1261176

[16] Rancati A, Angrigiani C, Hammond D, et al Preoperative digital mammography imaging in conservative mastectomy and immediate reconstruction. Gland Surg. 2016;5:9–14. doi: 10.3978/j.issn.2227-684X.2015.08.01 26855903 PMC4716857

[17] Frame J, Kamel D, Olivan M, Cintra H. The in vivo pericapsular tissue response to modern polyurethane breast implants. Aesthetic Plast Surg. 2015;39:713–723. doi: 10.1007/s00266-015-0550-4 26304599

[18] Siliprandi M, Vaccari S, Lisa AVE, et al Polyurethane-coated silicone breast implants: a viable option for primary augmentation mammoplasty: a systematic review and technical considerations. JPRAS Open. 2025;44:463–478. doi: 10.1016/j.jpra.2025.03.019 40452890 PMC12123325

[19] De Blacam C, Ogunleye AA, Momoh AO, et al High body mass Index and smoking predict morbidity in breast cancer surgery: a multivariate analysis of 26,988 patients from the national surgical quality improvement program database. Ann Surg. 2012;255:551–555. doi: 10.1097/SLA.0b013e318246c294 22330036

[20] Menon G, Alkabban FM, Ferguson T. Breast cancer. In: StatPearls. StatPearls Publishing; 2025. Accessed September 3, 2025. http://www.ncbi.nlm.nih.gov/books/NBK482286/29493913

[21] National Comprehensive Cancer Network . NCCN Clinical Practice Guidelines in Oncology (NCCN Guidelines^®^): Breast Cancer. April 2025. https://www.nccn.org/professionals/physician_gls/pdf/breast.pdf

[22] Panchal H, Matros E. Current trends in postmastectomy breast reconstruction. Plast Reconstr Surg. 2017;140:7S–13S. doi: 10.1097/PRS.0000000000003941 PMC5722225 29064917

[23] Malekpour M, Malekpour F, Wang HTH. Breast reconstruction: review of current autologous and implant-based techniques and long-term oncologic outcome. World J Clin Cases. 2023;11:2201–2212. doi: 10.12998/wjcc.v11.i10.2201 37122510 PMC10131028

[24] Regan JP, Casaubon JT. Breast reconstruction. In: StatPearls. StatPearls Publishing; 2025. Accessed September 3, 2025. http://www.ncbi.nlm.nih.gov/books/NBK470317/

[25] Gerber B, Marx M, Untch M, Faridi A. Breast reconstruction following cancer treatment. Dtsch Arztebl Int. 2015;112:593–600. doi: 10.3238/arztebl.2015.0593 26377531 PMC4577667

[26] Michelotti BF, Brooke S, Mesa J, et al Analysis of clinically significant seroma formation in breast reconstruction using acellular dermal grafts. Ann Plast Surg. 2013;71:274–277. doi: 10.1097/SAP.0b013e3182923dc9 23788150

[27] Chun YS, Verma K, Rosen H, et al Implant-based breast reconstruction using acellular dermal matrix and the risk of postoperative complications. Plast Reconstr Surg. 2010;125:429–436. doi: 10.1097/PRS.0b013e3181c82d90 20124828

[28] Wilkins EG, Hamill JB, Kim HM, et al Complications in postmastectomy breast reconstruction: one-year outcomes of the mastectomy reconstruction outcomes consortium (MROC) study. Ann Surg. 2018;267:164–170. doi: 10.1097/SLA.0000000000002033 27906762 PMC5904787

[29] Alderman AK, Wilkins EG, Kim HM, Lowery JC. Complications in postmastectomy breast reconstruction: two-year results of the Michigan breast reconstruction outcome study. Plast Reconstr Surg. 2002;109:2265–2274. doi: 10.1097/00006534-200206000-00015 12045548

[30] Washer LL, Gutowski K. Breast implant infections. Infect Dis Clin North Am. 2012;26:111–125. doi: 10.1016/j.idc.2011.09.003 22284379

[31] Catanuto G, Virzì D, Latino M, et al One-stage implant-based breast reconstruction with polyurethane-coated device: standardized assessment of outcomes. Aesthet Surg J. 2024;44:491–498. doi: 10.1093/asj/sjad301 37738139

[32] Duxbury PJ, Harvey JR. Systematic review of the effectiveness of polyurethane-coated compared with textured silicone implants in breast surgery. J Plast Reconstr Aesthet Surg. 2016;69:452–460. doi: 10.1016/j.bjps.2016.01.013 26887685

[33] Olsen MA . Hospital-associated costs due to surgical site infection after breast surgery. Arch Surg. 2008;143:53. doi: 10.1001/archsurg.2007.11 18209153

[34] Smith BD, Jiang J, Shih YC, et al Cost and complications of local therapies for early-stage breast cancer. J Natl Cancer Inst. 2017;109:djw178. doi: 10.1093/jnci/djw178 27678203 PMC6075189

[35] Gabriel A, Maxwell GP. Economic analysis based on the use of closed-incision negative-pressure therapy after postoperative breast reconstruction. Plast Reconstr Surg. 2019;143:36S–40S. doi: 10.1097/PRS.0000000000005311 30586102

[36] Olsen MA, Nickel KB, Fox IK, et al Incidence of surgical site infection following mastectomy with and without immediate reconstruction using private insurer claims data. Infect Control Hosp Epidemiol. 2015;36:907–914. doi: 10.1017/ice.2015.108 26036877 PMC4693606

[37] Jeevan R, Cromwell DA, Browne JP, et al Findings of a national comparative audit of mastectomy and breast reconstruction surgery in England. J Plast Reconstr Aesthet Surg. 2014;67:1333–1344. doi: 10.1016/j.bjps.2014.04.022 24908545

[38] Argenta LC, Morykwas MJ. Vacuum-assisted closure: a new method for wound control and treatment: clinical experience. Ann Plast Surg. 1997;38:563–576. discussion 577. doi: 10.1097/00000637-199706000-00002 9188971

[39] Lalezari S, Lee CJ, Borovikova AA, et al Deconstructing negative pressure wound therapy. Int Wound J. 2017;14:649–657. doi: 10.1111/iwj.12658 27681204 PMC7949947

[40] Shah A, Sumpio BJ, Tsay C, et al Incisional negative pressure wound therapy augments perfusion and improves wound healing in a swine model pilot study. Ann Plast Surg. 2019;82:S222–S227. doi: 10.1097/SAP.0000000000001842 30855392

[41] Glass GE, Murphy GF, Esmaeili A, Lai LM, Nanchahal J. Systematic review of molecular mechanism of action of negative-pressure wound therapy. Br J Surg. 2014;101:1627–1636. doi: 10.1002/bjs.9636 25294112

[42] Gabriel A, Sigalove S, Sigalove N, et al The impact of closed incision negative pressure therapy on postoperative breast reconstruction outcomes. Plast Reconstr Surg—Glob Open. 2018;6:e1880. doi: 10.1097/GOX.0000000000001880 30324063 PMC6181498

[43] Irwin GW, Boundouki G, Fakim B, et al Negative pressure wound therapy reduces wound breakdown and implant loss in prepectoral breast reconstruction. Plast Reconstr Surg—Glob Open. 2020;8:e2667. doi: 10.1097/GOX.0000000000002667 32309105 PMC7159936

[44] Annacontini L, Ciancio F, Parisi D, Innocenti A, Portincasa A. Management of nipple-areolar complex complications in skin-sparing mastectomy with prosthetic reconstruction A case report. Ann Ital Chir. 2016;5:1–3. 26829374

