# Supplementary material for: Can the Use of Closed Incision Negative Pressure Wound Therapy in Immediate Prepectoral Breast Reconstruction With Polyurethane-Coated Implants Reduce the Rate of Early Complications?: A Comparative Study
Source: Aesthet Surg J. 2025 Oct 23;46(10):1075–83. doi: 10.1093/asj/sjaf211 (PMC13577772; doi:10.1093/asj/sjaf211)
Supplement: Supplementary file 1 [file sjaf211_supplementary_data.docx]

**Supplemental Table 1**. Risk Factors Stratified By the Use of ciNPWT System

| Risk Factors | NO-ciNPWT (N = 363; %) | ciNPWT (N = 257; %) |
| --- | --- | --- |
| Patient-related   - Age > 65 - BMI ≥ 30 kg/m^2^ - Mastectomy volume > 500 gr - Type I diabetes - Type II diabetes - Vascular disorders - Hypertension - Immune disorders - Hypersensitivity syndrome - Smoking habits - Polypharmacy | 148 patients (40.77%)   - 22 patients (6.06%) - 14 patients (3.86%) - 22 patients (6.06%) - 0 patients (0%) - 3 patients (0.83%) - 0 patients (0%) - 31 patients (8.54%) - 4 patients (1.1%) - 5 patients (1.38%) - 42 patients (11.57%) - 5 patients (1.38%) | 131 patients (50.97%)   - 23 patients (8.95 %) - 15 patients (5.84%) - 25 patients (9.73%) - 0 patients (0%) - 3 patients (1.17%) - 0 patients (0%) - 15 patients (5.84%) - 4 patients (1.56%) - 2 patient (0.78%) - 42 patients (16.34%) - 2 patients (0.78%) |
| Cancer therapy-related   - Neo-adjuvant chemotherapy | - 75 patients (20.66%) | - 62 patients (24.12%) |
